# Supplementary material for: Antioxidant-Rich Clitoria ternatea Flower Extract Promotes Proliferation and Migration of Human Corneal Epithelial Cells
Source: Plants (Basel). 2025 Oct 20;14(20):3216. doi: 10.3390/plants14203216 (PMC12567149; doi:10.3390/plants14203216)
Supplement: Supplementary file 1 [file plants-14-03216-s001.zip › plants-3842979-supplementary.pdf]

Table S1. Compounds obtained through positive ionization mode.

No.	Retention Time (min)	Compound Name	Molecular Formula	[M+H]+ (m/z)	Peak Intensity (Absorbance)
1	1.088	Methylmethionine sulfonium	C6H14NO2S	164.0739	2769
	1.088	4-[(2-Methyl-3-furanyl)thio]-5-nonanone	C14H22O2S	255.1406	1439
	1.09	Imetit	C6H10N4S	193.0523	877
	1.091	Scutellarein 4'-methyl ether 7-glucuronide	C22H20O12	499.0827	555
	1.093	UDP-N-acetyl-2-amino-2-deoxy-D-glucuronate	C17H25N3O18P2	639.0929	698
2	2.275	Vernoflexuoside	C21H28O8	409.1862	849
	2.276	Thr-Phe Dipeptide	C13H18N2O4	267.1335	4753
	2.277	Isocarbostyril	C9H7NO	146.0601	2842
	2.277	Quinasetol	C11H9NO2	205.0967	75850
3	5.918	Delphinidin	C15H11O7	303.051	5809
	5.918	6-Hydroxycyanidin 3-glucoside	C21H21O12	465.1033	1537
	5.92	Delphinidin 3-neohesperidoside	C27H31O16	611.1597	7423
	5.921	Kaempferol 3-(2G-glucosylrutinoside)	C33H40O20	757.2182	27136
4	6.886	Pelargonidin 3-O-rutinoside 5-O-β-D-glucoside	C33H41O19	741.2219	7453
	6.886	Aurantinidin	C15H11O6	287.0546	97673
	6.887	Luteolin 7-rhamnosyl(1->6)galactoside	C27H30O15	595.165	115552
5	7.37	Luteolin 7-(6''-malonylneohesperidoside)	C30H32O18	681.1658	24363
	7.37	Cyanidin 3-(3''-malonylglucoside)	C24H23O14	535.1068	24746
	7.371	Aurantinidin	C15H11O6	287.0552	2692
6	10.447	11-Hydroperoxy-12,13-epoxy-9-octadecenoic acid	C18H32O5	346.258	26134
	10.45	Fucoxanthin	C42H58O6	681.4141	1066
	10.45	Ectocarpen	C11H16	149.1319	2611
	10.451	2-Methoxyestradiol-17β 3-sulfate	C19H26O6S	383.1518	5916
	10.451	4,4-Difluoropregn-5-ene-3,20-dione	C21H28F2O2	351.2129	14577
7	11.015	5,8,12-Trihydroxy-9-octadecenoic acid	C18H34O5	348.2737	45947
	11.016	9(S)-HpODE	C18H32O4	313.2374	8217

	11.018	6α-Hydroxycastasterone	C28H50O5	489.3557	932
8	13.894	Tiarubrin A	C13H8S2	250.9971	3583
9	16.058	(R)-Camphor	C10H16O	153.1276	2686
	16.059	11S-Hydroxytetradecanoic acid	C14H28O3	245.2109	4330
10	18.366	3-Butylidene-7-hydroxyphthalide	C12H12O3	205.0855	12111
	18.37	(-)-Jasmonoyl-L-isoleucine	C18H29NO4	324.2156	2414
11	19.61	4Z-Decenyl acetate	C12H22O2	199.1687	5158
12	22.604	Panaxydol linoleate	C35H54O3	540.4396	1160
	22.585	PtdIns-(3,4,5)-P3 (1,2-dipalmitoyl)-d62	C41H20D62O22P4	557.4141	486
	22.612	(10E)-19-Fluoro-1α,25-dihydroxyvitamin D3	C27H43FO3	473.282	898
13	24.115	Diisobutyl adipate	C14H26O4	259.1898	2454
	24.053	Panaxydol linoleate	C35H54O3	540.4403	717
	24.1	PE-Cer(d14:1(4E)/26:0)	C42H85N2O6P	783.5763	2548
	24.116	Docosanedioic acid	C22H42O4	371.3146	31608
	24.118	Dihydrofloroglucinol	C6H8O3	129.0552	2270
	24.123	Tetracosanedioic acid	C24H46O4	416.3723	3593
14	26.786	Methyl oxaloacetate	C5H6O5	184.9847	14250
	26.791	Erythrono-1,4-lactone	C4H6O4	156.9893	1884

*Note: m/z = mass-to-charge ratio; LC-MS/MS = Liquid Chromatography-Tandem Mass Spectrometry.

Table S2. Compounds obtained through negative ionization mode.

No.	Retention Time (min)	Compound Name	Molecular Formula	[M+H]+ (m/z)	Peak Intensity (Absorbance)
1	1.058	P1,P4-Bis(5'-xanthosyl) tetraphosphate	C20H26N8O23P4	982.9878	1604
2	6.262	Robinin	C33H40O19	739.2109	318562
3	6.699	Luteolin 7-rhamnosyl(1->6)galactoside	C27H30O15	593.1536	581217
4	15.233	2-Dodecylbenzenesulfonic acid	C18H30O3S	325.1847	5110
5	21.778	13,14-dihydro-15(R,S)-hydroxy-16,16-difluoro Prostaglandin E1-d4	C20H30D4F2O5	441.2622	2354
	21.778	4-Methyl-3-oxo-adipate	C7H10O5	173.045	5342

	21.778	Ramnetin 3'-glucuronide-3,5,4'-trisulfate	C22H20O22S3	844.9438	890
	21.78	Nodifloretin	C16H12O7	394.9762	1473
6	25.835	Carapanaubine	C23H28N2O6	427.1895	666
7	26.336	Dolastatin 16	C47H70N6O10	957.434	412
8	27.5	Not detected	-	-	-

*Note: m/z = mass-to-charge ratio; LC-MS/MS = Liquid Chromatography-Tandem Mass Spectrometry.
